# Design of Wideband High-Gain Patch Antenna Array for High-Temperature Applications

**DOI:** 10.3390/s23083821

**Published:** 2023-04-08

**Authors:** Ruibo Li, Peng Li, Paolo Rocca, Aarón Ángel Salas Sánchez, Liwei Song, Xinghua Li, Wanye Xu, Zijiao Fan

**Affiliations:** 1 Key Laboratory of Electronic Equipment Structure Design, Xidian University, Xi’an 710071, Chinapaolo.rocca@xidian.edu.cn (P.R.);; 2 DICAM—Department of Civil, Environmental, and Mechanical Engineering, Trento University, 38123 Trento, Italy

**Keywords:** antennas, patch array, wideband, high-gain, high-temperature

## Abstract

A low-profile, wideband, and high-gain antenna array, based on a novel double-H-shaped slot microstrip patch radiating element and robust against high temperature variations, is proposed in this work. The antenna element was designed to operate in the frequency range between 12 GHz and 18.25 GHz, with a 41.3% fractional bandwidth (FBW) and an obtained peak gain equal to 10.2 dBi. The planar array, characterized by a feed network with a flexible 1 to 16 power divider, comprised 4 × 4 antenna elements and generated a pattern with a peak gain of 19.1 dBi at 15.5 GHz. An antenna array prototype was fabricated, and the measurements showed good agreement with the numerical simulations as the manufactured antenna operated in the range of 11.4–17 GHz, with a 39.4% FBW, and the peak gain at 15.5 GHz was 18.7 dBi. The high-temperature simulated and experimental results, performed in a temperature chamber, demonstrated that the array performance was stable in a wide temperature range, from −50 °C to 150 °C.

## 1. Introduction

An antenna is, by definition, a sensor of EM (electromagnetic) waves; it plays an extremely important role in radar sensor [1], biomedical sensors [2], satellite information detection [3,4,5], remote control [6], and navigation [7,8]. Patch array antenna technology has dramatically improved over the last few decades due to the development of modern antenna technology having a low cost, a low profile, high reliability, and multiple functions. The main drawbacks of ordinary patch antennas are the narrow bandwidth, the low gain, and the sensitivity to the material parameters [9,10]. Especially for high-temperature environments, the temperature change can have a non-negligible impact on the material properties, thus causing frequency shifts and gain losses [11,12]. In this framework, this work aims to design a novel planar high-gain and wideband patch antenna array that works in the Ku-band, is robust against high temperature variations, and is suitable for EM wave sensing in a large temperature range. Therefore, the antenna design’s main challenge was achieving a suitable performance balance between antenna thickness, gain, and bandwidth.

Many effective methods have been proposed in the literature to increase the operation bandwidth of microstrip patch antennas. Among them, the use of parasitic patches has been considered as well as optimization of the antenna shape. More specifically, a patch antenna adopting a U-shaped slot-fed and stacked structure was proposed in [13], reaching a 2.58 GHz bandwidth (59.7% FBW) and a peak gain equal to 8 dBi. Other solutions have been considered using parasitic patches on the same substrate of the patch antenna [14,15]. Although this can effectively expand the bandwidth, a large 2D antenna aperture area is required. A different strategy considers the optimization of the shape of the slot used to feed the patch antennas. In this context, antennas using U-shaped slots have been adopted to increase the bandwidth [16,17,18], but in these cases, the gain did not exceed 5 dBi. Alternatively, spline-shaped profiles have also been exploited to model the contours of the patch radiating elements [19] and their optimization, yielded by acting on a limited set of geometric degrees of freedom. This has been addressed using advanced optimization methods [19,20].

Other methods have been investigated to improve the gain of the antenna element. In [21,22,23], the introduction of multiple short-circuit probes allowed high gain values between 8 dBi and 12 dBi to be achieved, with a bandwidth close to 500 MHz (13.4% FBW). In [24], using a feed network with four pin-diode switches, multiple working modes were excited on the antenna, including the common mode and differential feed schemes. This solution allowed for pattern reconfiguration, and a high gain pattern was obtained with an antenna bandwidth of 200 MHz (7% FBW). In [25,26,27], combining a short-circuit probe and multiport dual-polarized patches enabled the improvement of the antenna gain and the bandwidth, reaching values of 800 MHz (22% FBW). In [28], a novel method for removing part of the dielectric substrate was proposed, and the designed antenna was shown to achieve a gain of 7 dBi and 400 MHz bandwidth (10% FBW). In addition, electrically longer patch antennas provide higher gain, but narrower bands. In this framework, a long rectangular patch antenna with a single feed was proposed in [29], the gain of which reached 10.5 dBi. In [30], a structurally compact rectified antenna was fabricated, combining the characteristics of long patches and coplanar waveguides. In [31], by short-circuiting both ends of a long patch, various modes were excited to obtain an antenna gain to 9.7 dBi, and, jointly, a bandwidth of 13.2%. In [32], an ultra-wideband, high-gain, circularly polarized antenna was proposed, with FBW and peak gain equal to 49.8% and 8.5 dBi, respectively. More recently, many studies have considered multiport-fed dipole antennas to improve the antenna bandwidth and gain by increasing the radiation area of the dipole. In this framework, the antenna proposed in [33], based on the radiation mechanism of dipole and microstrip patch antenna, achieved high gain (8.9 dBi) and wideband (48% FBW) performance, but at a low frequency for application in this work.

This study proposes a novel double-H-shaped slot microstrip patch antenna, robust against high temperature variations, as a radiating element of a low-profile, wideband, and high-gain patch antenna array. In order to test the effectiveness of the proposed design, the antenna was fabricated and the prototype measured for comparison with the numerical results. The analysis of the temperature resistance was performed in a temperature chamber between −50 °C and 150 °C.

Accordingly, the remainder of this article is as follows. The elements of the antenna and antenna array designs are presented in Section 2. The prototype and the measurement results, also considering the analysis at high temperatures, are reported in Section 3. Eventually, some conclusions are given in Section 4.

## 2. Antenna Model and Design

### 2.1. Antenna Element Geometry

**Figure 1:**
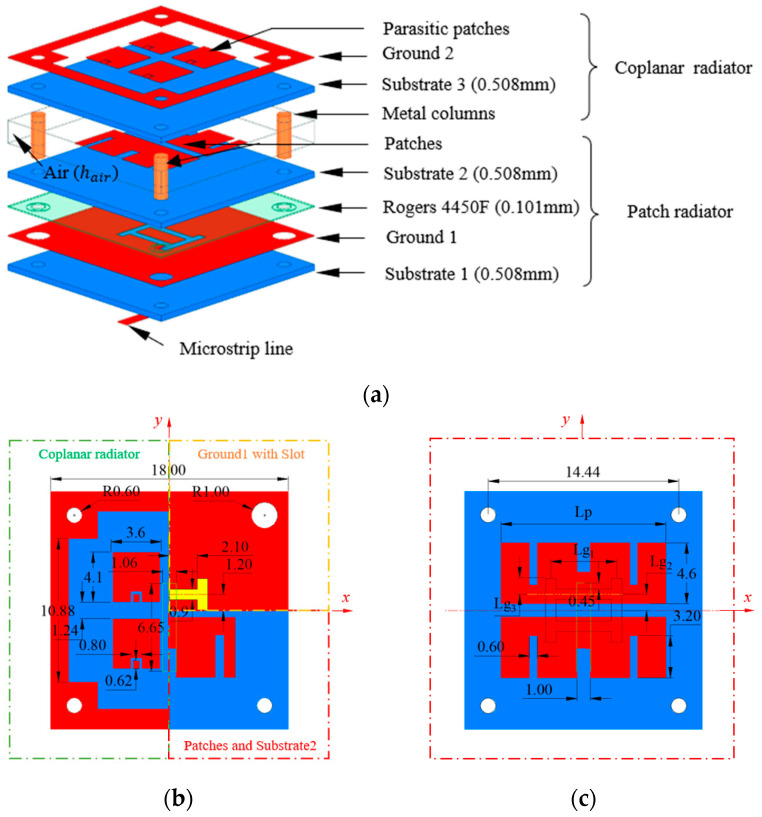
Antenna element structure: (**a**) 3D layout of the proposed antenna element; (**b**,**c**) parameter sizes, in millimeters, of the proposed antenna element ($L_{p}$ = 12.5 mm, $L_{g1}$ = 5 mm, $L_{g2}$ = $L_{g3}$ = 1.2 mm, $h_{air}$ = 2 mm); (**b**) ground 1 patches (patch radiator) and parasitic patches (coplanar radiator); (**c**) patches (patch radiator).

The proposed antenna element is shown in Figure 1, and comprises three parts. The first part is a patch radiator, which includes a pair of patches, two dielectric substrates (i.e., substrate 1 and substrate 2) characterized by a Rogers 4350B material (*ε_r_* = 3.66, tan *δ* = 0.004), and a ground plane (i.e., ground 1) with a double-H slot fed through a microstrip line. The second part is a coplanar radiator, which includes four parasitic patches, a ground plane (i.e., ground 2) connected with the ground plane of the first part by means of metallic pin columns, and a dielectric substrate (i.e., substrate 3). The third part, filling the space between the two radiators, consists of air (or foam).

### 2.2. Antenna Element Design

**Figure 2:**
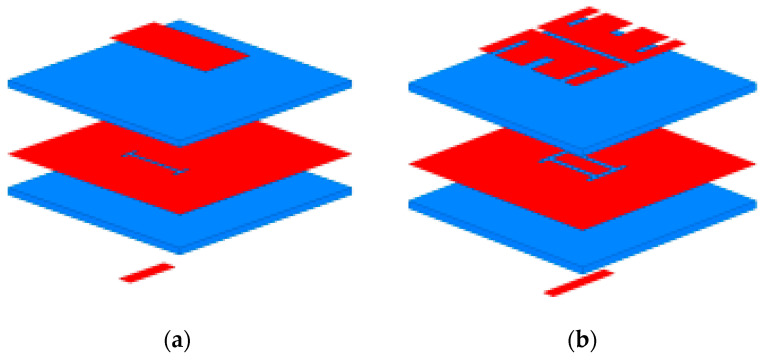

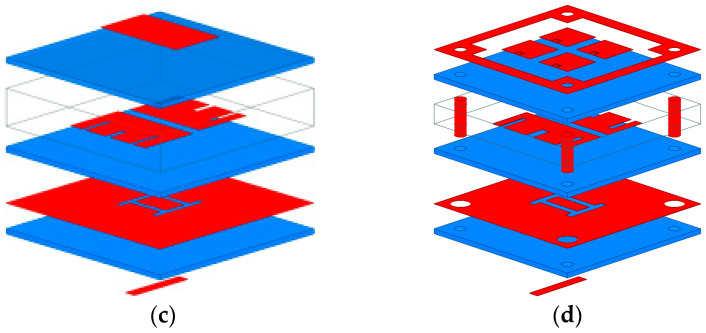
Design stages of the antenna element: (**a**) stage I, (**b**) stage II, (**c**) stage III, and (**d**) stage IV.

**Figure 3:**
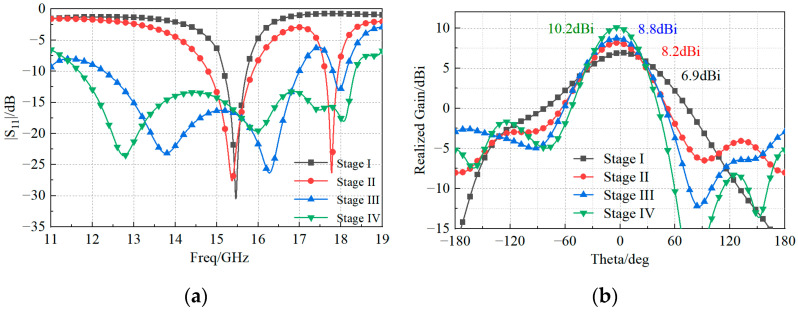
Simulated (**a**) magnitude of the reflection coefficient and (**b**) gain pattern at the four stages of the antenna design shown in Figure 2.

The antenna element proposed in this paper uses a novel double-H-shaped fed slot, which allows for the generation of multiple resonant modes thanks to the adopted double patch structure. More specifically, the coplanar parasitic radiator and the patch radiation structure form a resonant cavity, which reduces the Q-factor and increases the antenna’s bandwidth. As shown in Figure 2, the design of the proposed antenna element was carried out in four stages. For each stage, the antenna performance, and, more precisely, the magnitude of the reflection coefficient and the pattern gain, were simulated to demonstrate the obtained improvements (Figure 3).

In the first stage (Stage I), a patch antenna with a single H-shaped slot was considered. The resonant frequency of the microstrip patch working in the ${TM}_{10}$ mode was calculated as

(1) $$f_{{TM}_{mn}}=\frac{c}{2\pi \sqrt{\varepsilon_{r}}}\sqrt{{\left(\frac{m\pi }{W_{p}}\right)}^{2}+{\left(\frac{m\pi }{L_{p}}\right)}^{2}}$$

where $W_{p}$ and $L_{p}$ are the effective width and length, calculated according to [1]. The antenna bandwidth, computed as the frequency range for which the magnitude of the reflection coefficient is below −10 dB, is from 15.17 to 15.73 GHz, thus resulting in a 3.6% FBW and peak gain of 6.8 dBi at 15.5 GHz.

In the second stage (Stage II), two H-shaped feeding slots were considered in the ground plane. The geometrical parameters of the slot (Figure 1) satisfied the following condition:

(2) $$L_{g1}+L_{g2}+L_{g3}\approx \lambda_{g}$$

where the parameters $L_{g1}\approx \frac{\lambda_{g}}{2}$, $L_{g3}\approx \frac{\lambda_{g}}{4}$, $L_{g2}$ can be fine-tuned in order to optimize the impedance match. The antenna was characterized by two resonance frequencies at 15.5 GHz and 17.7 GHz (Figure 3a), with 7.1% FBW and 1.7% FBW, respectively. The peak gain of the pattern obtained at 15.5 GHz was 8.2 dBi (Figure 3b).

The third stage (Stage III) showed that the antenna’s bandwidth was greatly improved by adopting a stacked structure. Moreover, the peak gain increased to 8.8 dBi. The distance $h_{air}$ between the coplanar radiator and the patch radiator was calculated as

(3) $$h_{air}=\frac{c}{4\pi f}\left(\varphi_{1}+\varphi_{2}\right)+\frac{\lambda }{2}n,n=1,2,\ldots$$

where $\varphi_{1}$ and $\varphi_{2}$ are the reflection phase of the patch radiator and the coplanar radiator.

**Figure 4:**
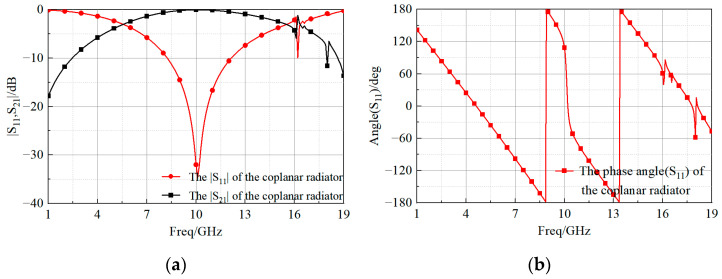
Values of the (**a**) magnitude and (**b**) phase of the reflection coefficient of the coplanar radiator.

The last stage (Stage IV) consisted of the final design, with a bandwidth of 12 GHz to 18.25 GHz (41.3% FBW) and a peak gain of 10.2 dBi. Figure 4 shows that the operating bandwidth of the coplanar radiator excited by FloquetPort was 8.25–12.14 GHz (38.2% FBW). When the patch radiator was used as its feed source, the coplanar radiator received near-field electromagnetic wave excitation formed by the patch, causing changes in its impedance bandwidth.

**Figure 5:**
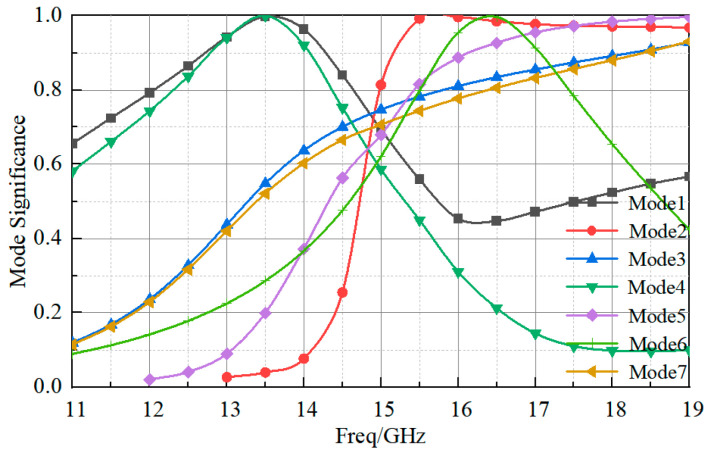
The mode significance of the coplanar radiator.

**Figure 6:**
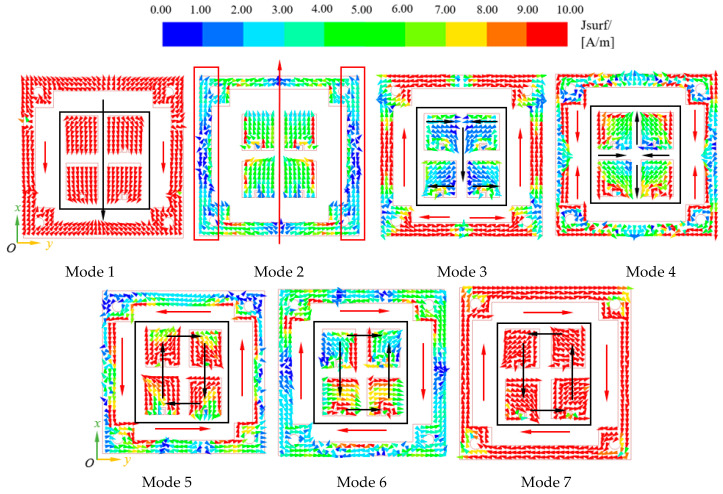
The current mode distribution of the coplanar radiator.

To maintain high gain over a wide bandwidth, the characteristic mode of the coplanar radiator was analyzed using HFSS to exploit the mode in order to generate a pattern with high gain. Figure 5 shows that the coplanar radiator was able to generate 7 modes, with corresponding mode significance |MS| > 0.707 in the range between 11 GHz and 20 GHz. The characteristic current corresponding to each mode is shown in Figure 6. The current of modes 1 and 2 was distributed unidirectionally along the *x*-axis and worked at different frequency bands. For Mode 1, the bandwidth of |MS| > 0.707 was between 12 GHz to 14.7 GHz, which can be considered as the lowest frequency part of the bandwidth in combination with the operating frequency band of the previous feed antenna. For Mode 2, the bandwidth of |MS| > 0.707 was 14.7 GHz to 20 GHz. By stimulating these two modes, the antenna can achieve a high gain in a wide band. In mode 3, the bandwidth of |MS| > 0.707 was between 14.5–20 GHz. The current of the parasitic patches and ground 2 was in the opposite direction along the x-axis and in the offset state. When using this mode, one needs to enhance or weaken a certain mode according to the mode of the patch radiator. The current components of the parasitic patch in mode 4 along the x and y axes were in the offset state, and it could not be used as the main radiation mode. Modes 5, 6, and 7 were unsuitable for linear polarization and high gain. The combination of modes 1, 2, and 3 of the coplanar radiator and the patch radiator enabled the overall structure to obtain a high gain and a high bandwidth.

**Figure 7:**
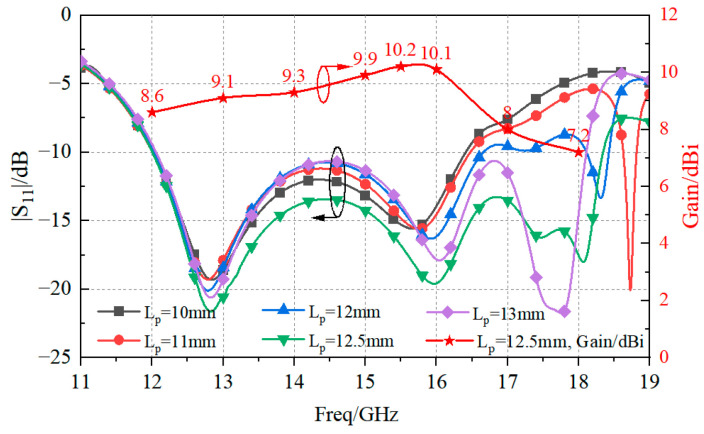
Numerical analysis of the magnitude of the reflection coefficient and peak gain versus *Lp*.

Indeed, the antenna resonated in the frequency range between 12 GHz and 18.25 GHz, with a 41.3% FBW, and the peak gain was equal to 10.2 dBi at 15.5 GHz. In order to achieve such a performance, the value of the parameter *Lp* (i.e., the length of radiation patches) was properly tuned. As is evident from Figure 7, the antenna element reached the widest bandwidth when *Lp* = 12.5 mm.

**Figure 8:**
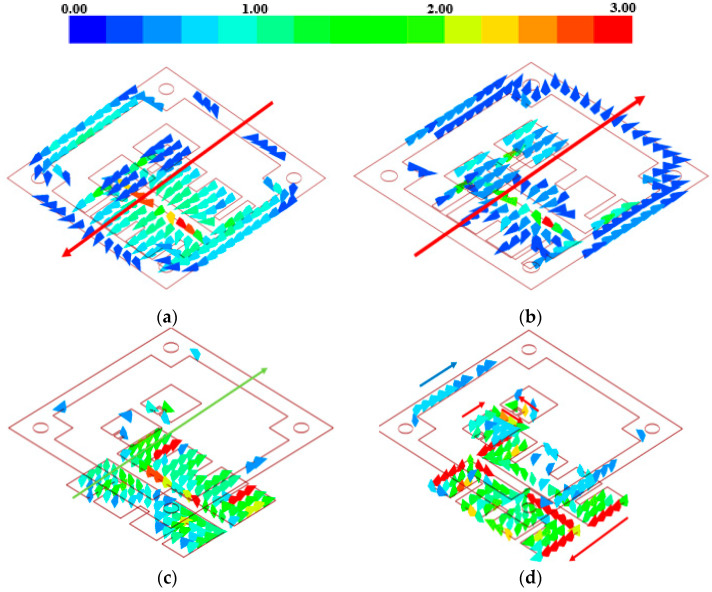
Current distribution on the antenna element at (**a**) 13 GHz, (**b**) 16 GHz, (**c**) 17.5 GHz, and (**d**) 18 GHz.

**Figure 9:**
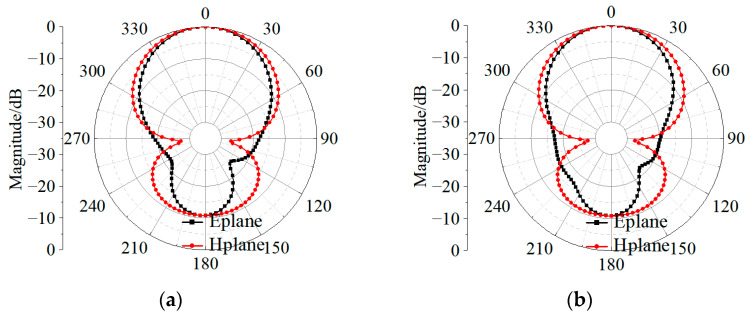

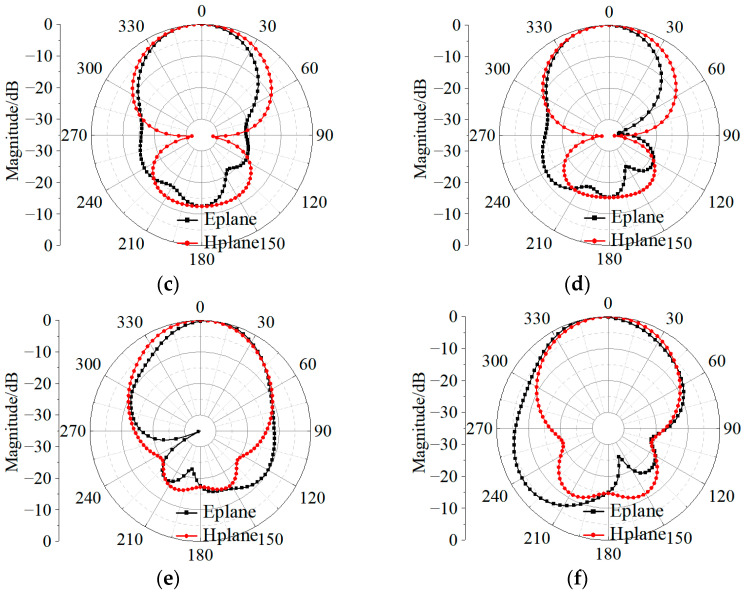
Radiation patterns of the proposed antenna at (**a**) 13 GHz, (**b**) 14 GHz, (**c**) 15 GHz, (**d**) 16 GHz, (**e**) 17.5 GHz, and (**f**) 18 GHz.

The working principle of the proposed antenna element can be described as follows. At the frequency f = 13 GHz, the radiation patch worked as shown in Figure 8a, and an overall downward current was induced on the metallic patches. The edge of the ground plane in the coplanar radiator (ground 2) supported a current induced in the same direction, thus resulting in the superposition of two radiation modes. The pattern gain, shown in Figure 9a, reached a peak value of 9.1 dBi. At f = 16 GHz, the radiation patch worked as shown in Figure 8b, and an overall upward current was induced on the patch. The parasitic patches and the ground plane in the coplanar radiator supported an induced current in the same direction. The corresponding pattern is shown, in this case, in Figure 9d, and the achieved peak gain was equal to 10.1 dBi. Differently, at f = 17.5 GHz, the current induced on the radiation patch was mainly concentrated near the gaps, as shown in Figure 8c. There was no current induced in the coplanar radiator, which became a transmission surface. The pattern obtained in this case is shown in Figure 9e. At f = 18 GHz, the current induced on the radiation patch was mainly concentrated at the narrow edge, as shown in Figure 8d, and the parasitic patches in the coplanar radiator also supported a current mode. The peak gain resulted to be equal to 7.2 dBi, and the corresponding pattern is reported in Figure 9d.

For the sake of comparison, Table 1 reports the principal geometrical and electrical features of the proposed antenna solution as compared to other patch antennas previously published in the literature. Compared with the antenna element in [1,2], which adopted a large number of FSS parasitic structures to improve its bandwidth and gain, our element reached a wider bandwidth, with smaller floor space and good performance in terms of gain. Compared with the element in [8], which was cavity-backed to improve the gain, our work achieved a wider bandwidth and a higher gain. Compared with the antenna element in [13], which had a traditional stacked structure, a single H-slot, and air dielectric substrates, our antenna element had a higher gain, although the FBW was smaller since the substrate had higher permittivity. The antenna element in [32,33], which adopted a double-Y-shaped slot and microstrip dipole antenna, respectively, still had a wider FBW, but less peak gain. In addition, the antenna element in [34] needed to match a more complex feeding structure. As compared to the antennas in [31,34,35], characterized by a rectangular patch [31]; a single-slot, multilayer parasitic structure [34]; and a planar magneto-electric dipole stack structure [35], our solution achieved a wider bandwidth and a greater gain. Accordingly, in comparison with previous works, the antenna proposed in this paper guaranteed a good trade-off between bandwidth, gain, and antenna size.

### 2.3. 4 × 4 Antenna Array Design

**Figure 10:**
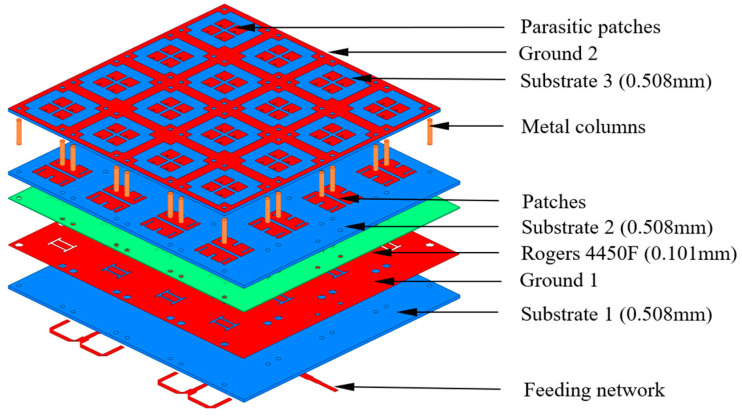
Structure of the proposed 4 × 4 antenna array.

**Figure 11:**
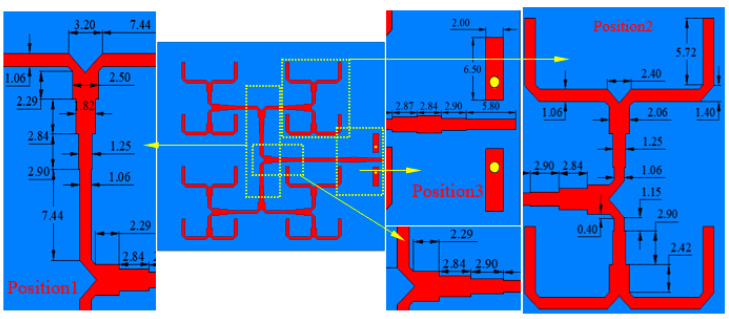
Structure of the corporate feeding network.

In addition to the antenna element, the design of a 4 × 4 antenna array, shown in Figure 10, was carried out in this work. Towards this end, the inter-element spacing was set according to the antenna element length (0.9λ < λ), and an equal power division (1 to 16 divider) in a corporate feed network was properly designed. Figure 11 illustrates the geometry of the feeding network and of the 1-to-16 power divider.

**Figure 12:**
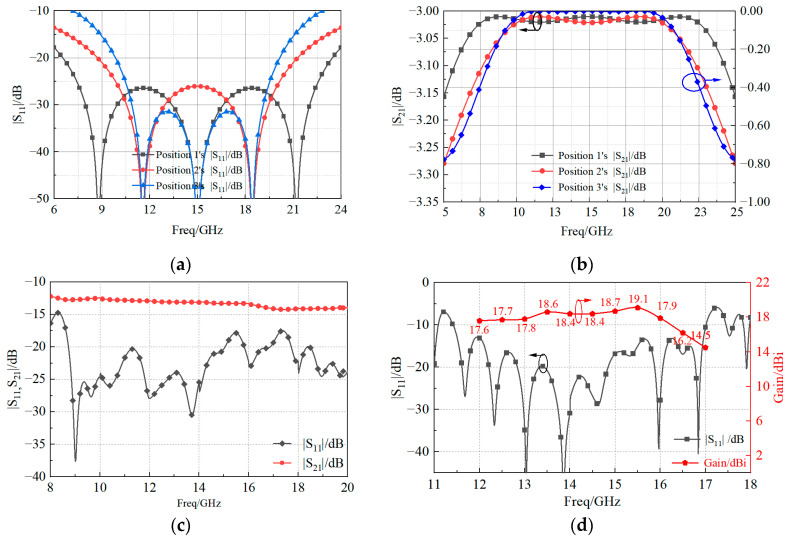
Simulated magnitude of the (**a**) reflection and (**b**) transmission coefficients of position 1, position 2, and position 3; the (**c**) whole $\left|S_{11},S_{21}\right|$ of the feeding network; and the (**d**) reflection coefficient and gain of the proposed antenna array.

The simulated reflection and transmission coefficients at positions 1, 2, and 3 of Figure 11 are shown in Figure 12a and Figure 12b, respectively. In the frequency range of [10,11,12,13,14,15,16,17,18,19,20] GHz, the reflection coefficient obtained in the three positions was lower than −25 dB. The transmission coefficients of the one-to-two T-type impedance transformation section at position 1 and 2 were higher than −3.1 dB, and the impedance compensation transformation section at position 3 was higher than −0.2 dB, thus implying reduced losses along the feeding line. The plot of the simulated reflection and transmission coefficients of the feed network, shown in Figure 12c, demonstrated a good matching ability with |*S*_11_| values below −15 dB from 9 to 20 GHz. As for the array, the values of the simulated reflection coefficient and of the peak gain given in Figure 12d show that the antenna operated (i.e., |*S*_11_| < −10 dB) from 11.4 GHz to 17 GHz, and the maximum gain was 19.1 dBi.

## 3. Experimental Measurements and Discussion

### 3.1. Measurement at Normal Temperature

**Figure 13:**
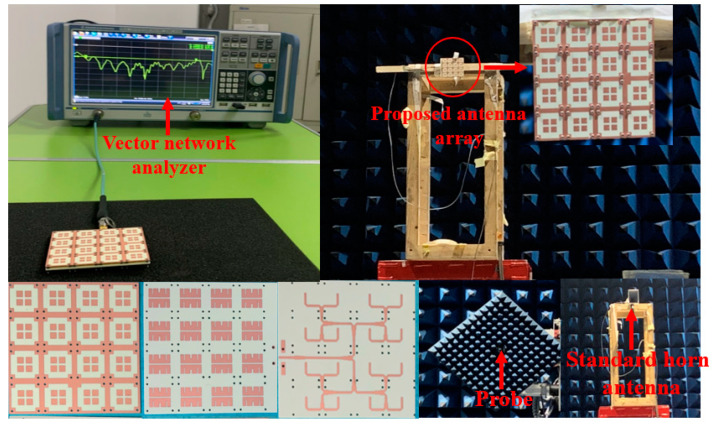
Photographs of the fabricated prototype and measurement setup.

**Figure 14:**
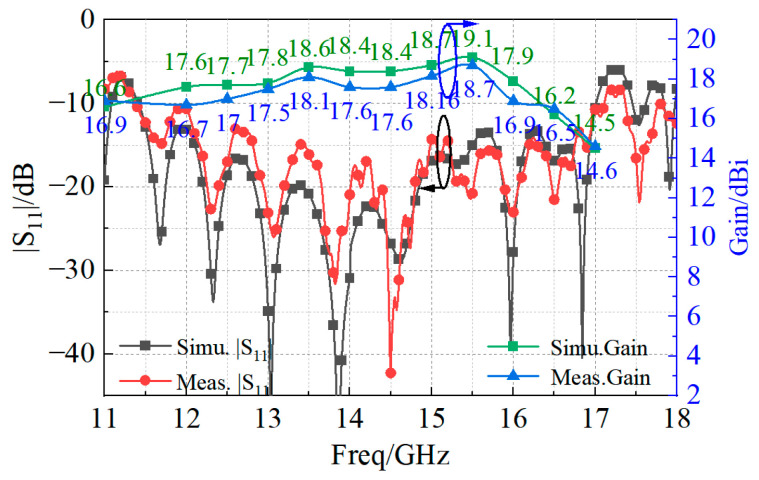
Comparison between simulated and measured reflection coefficient magnitude.

**Figure 15:**
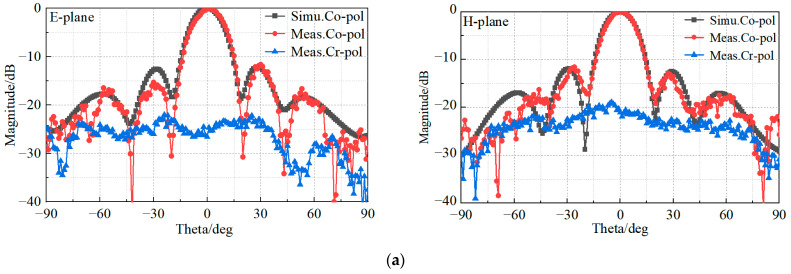

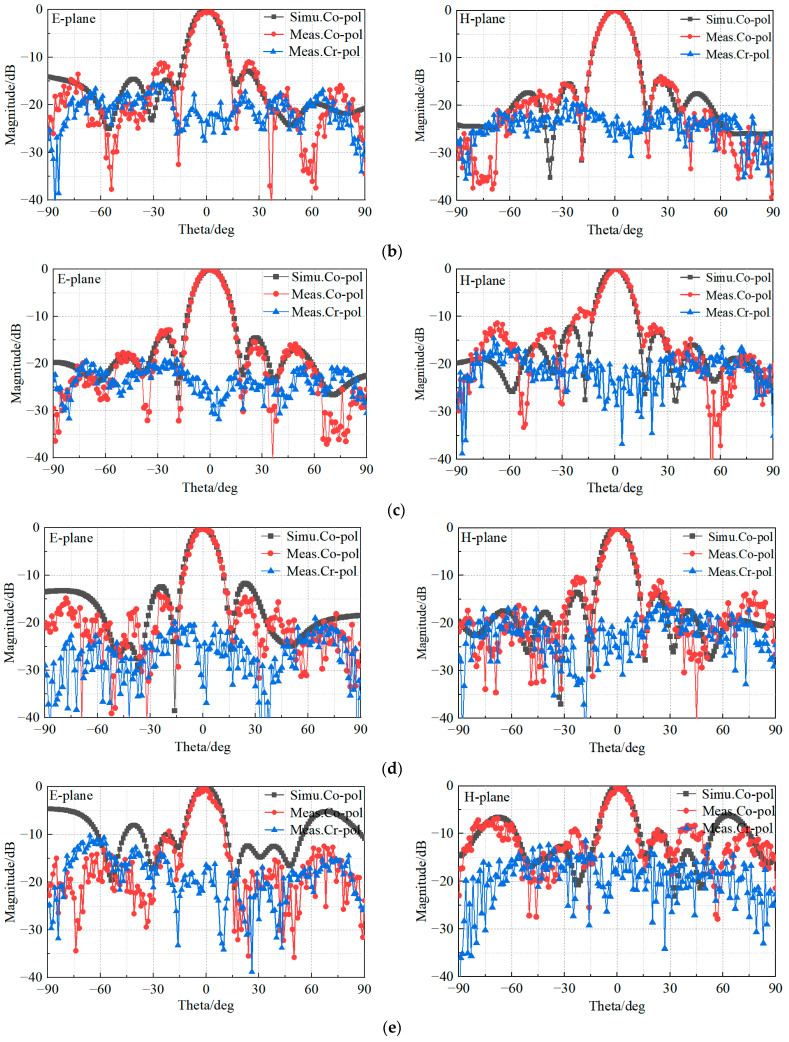
Measured and simulated radiation patterns of the proposed 4 × 4 wideband antenna array at (**a**) 12.5 GHz, (**b**) 13.5 GHz, (**c**) 14.5 GHz, (**d**) 15.5 GHz, and (**e**) 16.5 GHz.

The prototype of the proposed 4 × 4 patch antenna array is shown in Figure 13. The patch radiator and the coplanar radiator (Figure 1a) were assembled by stainless steel screws. The final dimensions of the fabricated prototype resulted as 78 × 78 × 3.7 ${mm}^{3}$. To measure the reflection coefficient, a ROHDE and SCHWARZ vector network analyzer was used. The radiation patterns were instead measured in an anechoic far-field chamber at Space Star Technology Co., Ltd., in Xi’an, China. The gain was measured using the gain comparison method with standard gain horns, and the gain measurement uncertainty ranged between ±0.5 dB according to the specification of the anechoic far-field chamber. The values of the simulated and measured magnitude of the reflection coefficient, as well as the peak gain, are shown in Figure 14. The operation bandwidth resulted to be almost 5.6 GHz, with a FBW = 39.4%, while the peak gain at 15.5 GHz was 18.7 dBi, only 0.4 dB below the simulated value. In addition, the gain measurements showed good agreement with the simulated values, since the deviation was smaller than 0.5 dB in the range of 13–14 GHz and 15–17 GHz, and 1 dB in the range of 11.4–13 GHz and 14–15 GHz. Moreover, the power pattern measured in the E- and H-plane at 12.5, 13.5, 15.5, and 16.5 GHz agreed well with the simulated results, as shown in Figure 15. From the analysis of the pattern at 12.5, 13.5, and 15.5 GHz, it resulted that the relative sidelobe level was below −11 dB. As for the cross-polarization level, it was always below −15 dB, and below −20 dB in the angular range ± 20°. At 15.5 GHz, the mismatch between sidelobe levels in the E- and H-planes was about ±2 dB. At 16.5 GHz, the array antenna had obvious side radiation, which reduced its gain. In addition, the measured patterns had lower relative sidelobe levels as compared to the simulated results. We summarize the reasons for the differences between the simulation and the experiment as follows: First, it is difficult to avoid errors introduced during antenna processing. Furthermore, the antenna is affected by tools such as clamps during testing, which affects its side radiation. Moreover, errors in antenna sampling and turntable position during testing can also affect the antenna’s performance. In addition, if the antenna needs to be used in situations with high sidelobe requirements, the operating frequency band should be controlled within the range of 12 GHz to 15.5 GHz.

### 3.2. Measurement in High-Low Temperature Chamber

**Figure 16:**
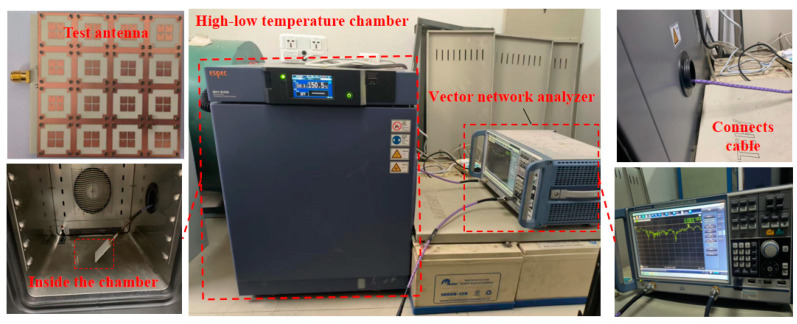
Measurement setup in the high–low temperature chamber.

In order to verify the performance of the proposed antenna in high-temperature environments, the measurement of the reflection coefficient was carried out in a high–low temperature chamber, as shown in Figure 16.

**Figure 17:**
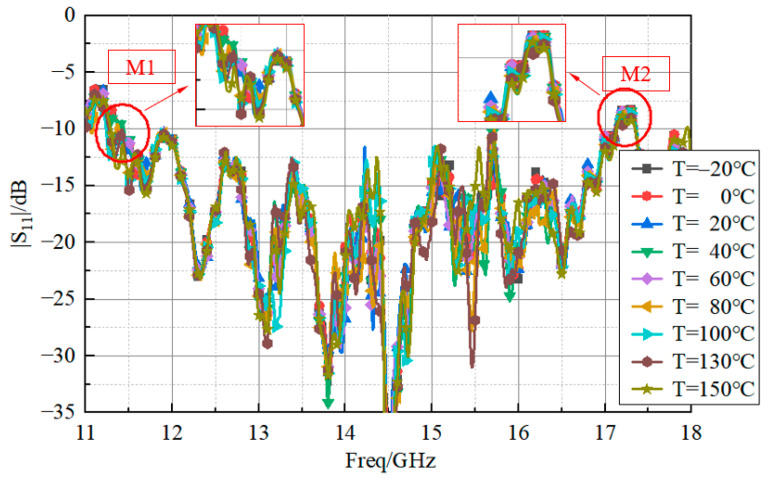
Measured reflection coefficient at nine different temperatures.

More specifically, an ESPEC (SH-642) high–low temperature chamber and a vector network analyzer were used. The measurement considered nine different temperatures, namely, 20 °C, −20 °C, 0 °C, 40 °C, 60 °C, 80 °C, 100 °C, 130 °C, and 150 °C. The behavior of the magnitude of the reflection coefficient versus the frequency is shown in Figure 17.

**Figure 18:**
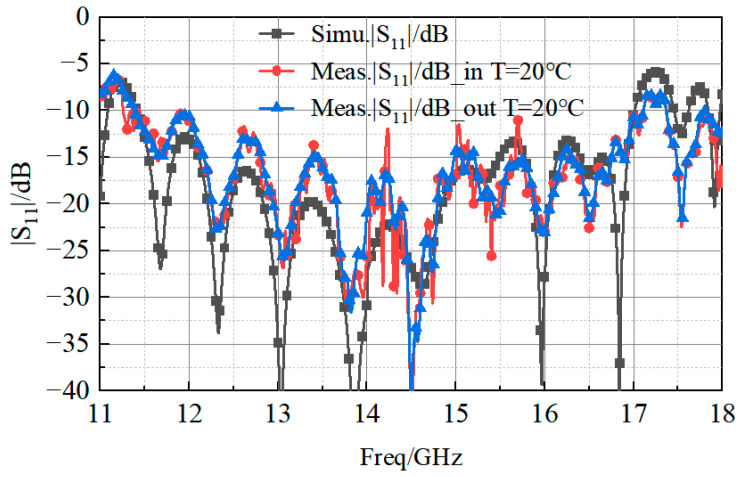
Measured reflection coefficient at a temperature of 20 °C inside and outside the high–low temperature chamber.

The values of the minimum frequency (M1), the maximum frequency (M2), and the bandwidth of the curves shown in Figure 17 are reported in Table 2. It is clear that the proposed antenna had good bandwidth stability in the temperature range from −20 °C to 150 °C, as the reflection coefficient showed marginal variations during the high–low temperature tests. For the sake of completeness, Figure 18 reports the values of the reflection coefficients, measured at a temperature of 20 °C, with the antenna in the chamber (curve “20 °C_in”) and outside the chamber (curve “20 °C_out”).

**Figure 19:**
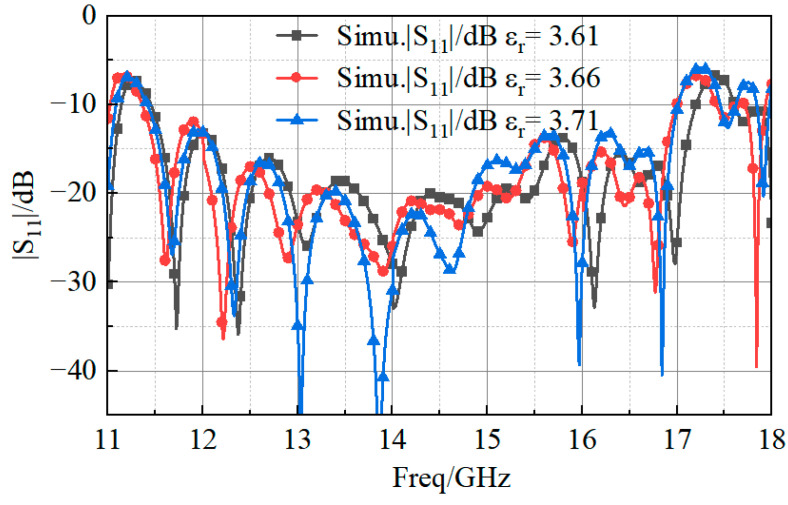
Simulated reflection coefficient magnitude values.

According to the datasheet on the substrate material, the expected variation of the permittivity in the temperature range between −50 °C and 150 °C was about 3.66 ± 0.05. Therefore, the impact on the magnitude of the reflection coefficients was also simulated (Figure 19). Like in Table 2, the corresponding values of the minimum frequency (M1), the maximum frequency (M2), and the bandwidth reported in Table 3 demonstrate that there was good agreement with the experimental measurements.

**Figure 20:**
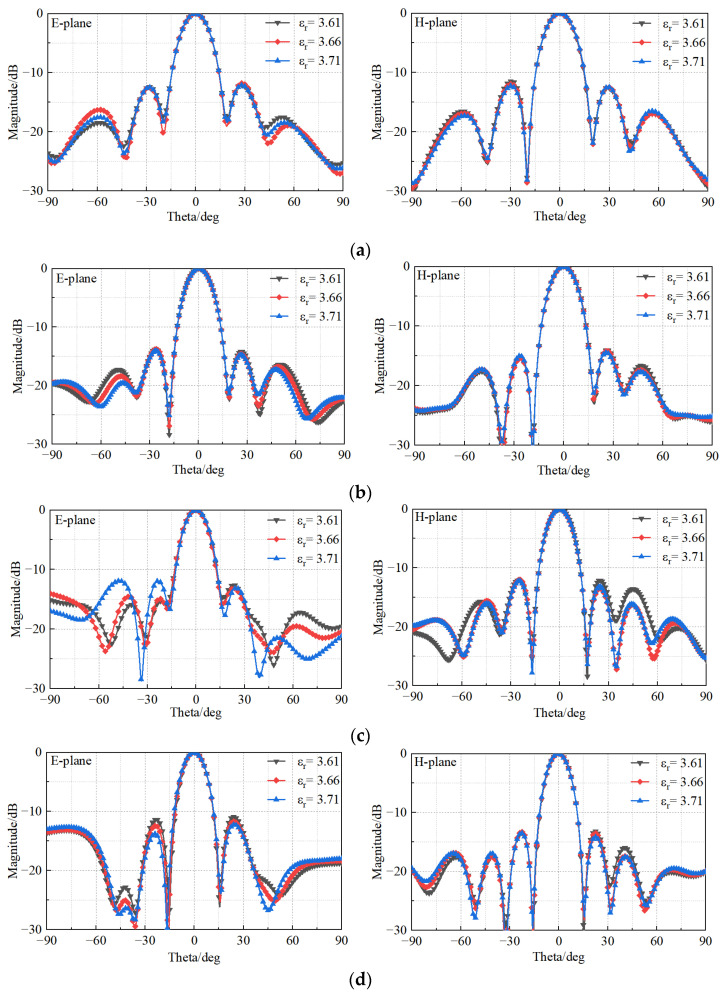

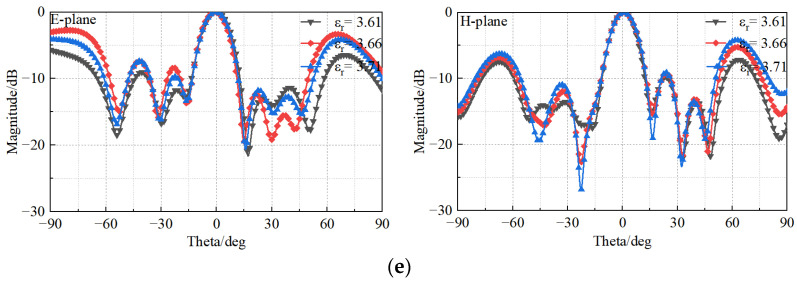
Simulated radiation patterns of the antenna in a high–low temperature chamber at (**a**) 12.5 GHz, (**b**) 13.5 GHz, (**c**) 14.5 GHz, (**d**) 15.5 GHz, and (**e**) 16.5 GHz.

Moreover, although the power pattern could not be measured in the high–low temperature chamber, it has been simulated when changing the material property, and the results in Figure 20 confirm the expected stability of the pattern.

## 4. Conclusions

The design of a low-profile, wide-band, and high-gain 4 × 4 antenna array based on a novel double-H-shaped slot microstrip patch radiating element, robust against high temperature variations, was presented. The corresponding prototype was fabricated, and the measurement results demonstrated that the antenna array reached a 5.6 GHz bandwidth (39.4% FBW, Ku-band) and a maximum gain of 18.7 dBi. The test and simulation results also indicate that the proposed antenna array had good stability in a temperature range from −50 °C to 150 °C, and that, therefore, the antenna has good potential to be applied in higher-temperature environments.

## Figures and Tables

**Table 1:** Comparison with benchmark patch antennas.

Ref	Dimensions $\left(\lambda_{0}\times \lambda_{0}\times \lambda_{0}\right)$	Permittivity $\left(\varepsilon_{r}\right)$	Bandwidth, FBW (GHz, %)	Peak Gain (dBi)	Number of Ports
[3]	4.8 × 4.8 × 0.63	2.2	8.3–11.25, 29.5%	10.4	1
[4]	1.48 × 1.48 × 0.04	3	8.17–9.61, 16.2%	8.9	1
[8]	0.7 × 0.7 × 0.13	2.2	25.58–27.04, 5.2%	7.8	1
[13]	/	1.1	3.27–6, 59.7%	8	1
[31]	1.3 × 0.7 × 0.04	2.2	5.13–5.85, 13.1%	9.7	1
[32]	0.4 × 0.4 × 0.23	2.65	3.6–6, 49.8%	8.5	1
[33]	0.78 × 0.78 × 0.18	4.4	1.68–2.75, 48%	8.9	2
[34]	0.5 × 0.5 × 0.25	3.2	6.5–10.2, 37%	7.8	1
[35]	0.96 × 0.96 × 0.14	2.5	5.2–5.9, 12.6%	9.6	1
Present	0.9 × 0.9 × 0.18	3.66	12–18.25, 41.3%	10.2	1

**Table 2:** Measurement of the antenna bandwidth at different temperatures.

Temperature (°C)	M1 (GHz)	M2 (GHz)	Bandwidth (GHz)
−20	11.39	17.04	5.65
0	11.38	17.02	5.64
20	11.27	17.01	5.74
40	11.44	17.12	5.68
60	11.46	17.10	5.64
80	11.38	17.12	5.74
100	11.40	17.12	5.72
130	11.44	17.10	5.66

**Table 3:** Simulation of antenna bandwidth at high and low temperatures.

Permittivity	M1 (GHz)	M2 (GHz)	Bandwidth (GHz)
3.61	11.45	17.20	5.75
3.66	11.4	17	5.6
3.71	11.36	17	5.64

## Data Availability

Not applicable.
